# Lifestyle choices and mental health: a representative population survey

**DOI:** 10.1186/s40359-014-0055-y

**Published:** 2014-12-23

**Authors:** Julia Velten, Kristen L Lavallee, Saskia Scholten, Andrea Hans Meyer, Xiao-Chi Zhang, Silvia Schneider, Jürgen Margraf

**Affiliations:** 1 Faculty of Psychology, Department of Clinical Psychology and Psychotherapy, Mental Health Research and Treatment Center, Ruhr-Universität Bochum, Massenbergstrasse 9-13, D-44787 Bochum, Germany; 2 Department of Psychology, University of Basel, Basel, Switzerland

**Keywords:** Lifestyle, Mental health, Depression, Anxiety, Physical activity, Smoking, Alcohol, Obesity

## Abstract

**Background:**

Identifying healthy lifestyle behaviours that promote psychological wellbeing is crucial to preventing mental disorders. The aim of the current study was to evaluate the individual and combined associations between different aspects of everyday life and mental health within a representative community sample in Germany.

**Method:**

The study was conducted in 2012/2013 and included 7,937 participants representative of the German population. Lifestyle factors were assessed via self-report and included frequency of physical and mental activity, alcohol consumption, smoking, body mass index as well as circadian and social regularity. Outcome variables were depression, anxiety, stress and satisfaction with life.

**Results:**

All lifestyle factors were associated with the mental health outcomes. Better mental health was linked to higher frequency of physical and mental activity, moderate alcohol consumption (i.e. not increased or no alcohol consumption), non-smoking, a body mass index within the range of normal to overweight (i.e. not underweight or obese) and a regular life rhythm. The more healthy lifestyle choices an individual makes, the higher life satisfaction and lower psychological distress he or she tends to have.

**Conclusions:**

The current study underlines the importance of healthy lifestyle choices in respect to psychological wellbeing.

## Background

Mental health is increasingly recognized as a critical component of general public health (Herrman et al. 2005). Every year, more than 38% of the population within the European Union suffers from at least one mental disorder. Both direct health care costs as well as non-medical and indirect costs are immense, as mental and neurological disorders led to total costs of €798 billion in Europe in 2010. The most expensive mental disorders are mood (€113.4 billion), psychotic (€94.0 billion), and anxiety disorders (€74.4 billion) with unipolar depression being the most burdensome in terms of years of lost productivity due to disability or premature mortality (Olesen et al. 2012). Indeed, the need for health promotion, prevention and treatment programs for mental disorders is arguably the primary health challenge of the 21st century in Europe (Wittchen et al. 2011). Identifying risk and protective lifestyle factors that promote mental health is thus crucial to reducing the burden of mental disorders.

## Lifestyle behaviours and health

Mental health is influenced not only by trait markers, general living conditions and major life events, but also, as increasing evidence indicates, by simple everyday behaviours that can be altered by an individual. Prospective studies consistently find a bidirectional relationship between various lifestyle factors and physical as well as mental health, with important health improvements and wellbeing following relatively small changes in lifestyle (Jonsdottir et al. 2010; Xu et al. 2010).

Various lifestyle choices are known promoters of physical health, including engaging in sports or moderate to rigorous physical activity (Fogelholm 2010), participating in cultural or mental activities, like singing in a choir or reading a book (Bygren et al. 1996; Bygren et al. 2009), refraining from smoking (Schane et al. 2010), practicing moderation in alcohol consumption (Ronksley et al. 2011), maintaining a body mass index (BMI) within the range of normal weight (Harriss et al. 2009b), and eating a healthy diet (Scarborough et al. 2012). A robust relationship between these lifestyle choices and various somatic diseases, including cancer (Harriss et al. 2009a), heart disease (Sattelmair et al. 2011), or stroke (He et al. 2006) is well documented.

Evidence indicates that such lifestyle factors also have a positive effect on the psychological domain, reducing depression as well as anxiety (Scott et al. 2008; Xu et al. 2010), increasing life satisfaction (Headey et al. 2013) and self-perceived general mental health (Chaney et al. 2007; Hamer et al. 2009; Rohrer et al. 2005). A recent review by Mammen and Faulkner (2013) analysed 30 prospective longitudinal studies and identified physical activity as an important protective factor in reducing the risk of developing depression. Another systematic review acknowledged positive effects of exercise training interventions on reducing symptoms of anxiety in patients with chronic illnesses. Systematic aggregation of 40 treatment studies estimated the anxiety reduction with an average effect size of *d* = 0.29 for the training condition as compared with the controls without exercise training (Herring et al. 2010). Cuypers et al. (2012) reported a small, but positive effect of cultural or creative activities on various mental health outcomes, including depression, anxiety and life satisfaction in both men and women. As in the domain of physical health outcomes, smoking has also been identified as a risk factor for psychological distress (Kinnunen et al. 2006; Lien et al. 2009). The relationship between alcohol consumption and psychological distress is still controversial. While some studies identify a nonlinear relationship, with elevated risks for depression and anxiety for abstainers and heavy drinkers as compared to light/moderate drinkers (Rodgers et al. 2000), other studies did not find any meaningful correlation between alcohol consumption and symptoms of psychological distress (Xu et al. 2010). The research regarding the relationship between body mass index (BMI) and mental health is also contradictory. Data from 46,704 participants in the South Australian Monitoring and Surveillance System, which measures nationwide trends in risk factors and chronic diseases, indicated a nonlinear relationship between BMI and mental health, with greater odds of mental health problems only in obese women (Kelly et al. 2011). Other studies suggested elevated risks for mental disorders for young women with obesity (Becker et al. 2001) or described underweight individuals (Molarius et al. 2009) as an additional risk group. In addition to the above mentioned lifestyle factors, the influence of circadian and social rhythms on mental health are currently under investigation. The association between various mental disorders and disturbances of circadian rhythms is also documented, especially for schizophrenia, bipolar disorder and depression (Jagannath et al. 2013). Disruptions of circadian rhythms may indeed trigger or exaggerate episodes of mania (McClung 2007) and first evidence suggests that cognitive behavioural treatment of insomnia may improve psychotic symptoms in individuals with persistent delusions (Myers et al. 2011). Although the exact mechanisms are still unclear, there is evidence that the circadian system is also an important regulator of other vital functions and influences one’s capacity for mood regulation (McClung 2013). Additionally, irregular social rhythms, which include social contacts, are also associated with mood disorders. A recent study indicated that elderly patients with major depressive disorder exhibit a lower regularity in their social rhythms compared to healthy controls (Lieverse et al. 2013). Although the understanding of the bidirectional relations between affective disorders and life rhythm is still preliminary, research indicates that social and circadian rhythms play an important role in understanding mood disorders (Grandin et al. 2006).

## Lifestyle composite scores

Healthy and unhealthy lifestyle behaviours tend to occur in clusters (Conry et al. 2011). A healthy way of life can be characterized as an accumulation of multiple healthy lifestyle choices. Therefore, research has recently been approaching lifestyle with a more holistic view and seeks to evaluate the cumulative effects of protective lifestyle behaviours on health outcomes.

An evaluation of data from the EPIC-Norfolk Prospective Population Study indicated that the presence of a combination of four important healthy lifestyle behaviours decreased mortality substantially when compared with an absence of these behaviours (Khaw et al. 2008). Healthy lifestyle behaviours in this study included non-smoking, at least 30 minutes of daily physical activity, moderate or no alcohol intake and at least sufficient fruit/vegetable intake (measured via plasma vitamin C levels). With rising number of healthy behaviours, a corresponding reduced mortality risk over the evaluated period of eleven years was observed. For example, individuals that engaged in all four health behaviours had a 4-fold reduced mortality risk – equivalent to 14 years longer life expectancy – as compared to persons who did not meet the threshold on any of these behaviours (Khaw et al. 2008). The same pattern of results was found for the relation between the number of health behaviours and stroke. These four behaviours predicted more than a twofold difference in incidence of stroke in the same study population (Myint et al. 2009).

A similar protective lifestyle behaviour (PLB) score was used to evaluate the effect of lifestyle on self-perceived overall health and depression (Harrington et al. 2010). This PLB score also included being physically active, non-smoking, moderate alcohol consumption and adequate fruit and vegetable intake. A higher number of PLBs was associated with better perceived overall health and greater mental health outcomes. Individuals displaying no PLBs had a more than four times higher likelihood of suffering from a major depressive disorder and a seven times lower chance of perceiving their health as excellent/very good than subjects with four PLBs.

While there is evidence that physical health and depressive symptoms improve with a rising number of PLBs, there is a lack of literature concerning the effect of a combination of PLBs on life satisfaction or on other psychological syndromes like anxiety or stress. Research has focused mainly on certain lifestyle factors, such as physical activity, smoking or alcohol consumption, while other aspects of everyday life have not been researched satisfactorily. Social and circadian rhythm, although known predictors or mental health, have not been investigated as part of a PLB score and although research revealed a dose–response relationship between the number of cultural and creative activities and mental health (Cuypers et al. 2012), this health-related lifestyle has not yet been investigated in combination with other lifestyle factors.

## The present study

The aim of the present study, therefore, was to extend the work on the impact of major lifestyle factors to a broad spectrum of aspects of mental health and to analyse the individual and combined associations between lifestyle health behaviours and psychological distress or wellbeing. The study included major lifestyle factors previously shown to have an effect on physical health and depression, including smoking, alcohol drinking frequency, physical activity and body-mass-index as predictors of mental health in a representative community sample. Additionally, we included mental/cultural activity and circadian and social rhythms, previously under-investigated in population-based surveys, and aimed to investigate their unique contributions in predicting mental wellbeing in the general public. Following the approach of Harrington et al. (2010) and Khaw et al. (2008), we sought to examine the combined associations of the examined lifestyle behaviours with mental health and life satisfaction.

Positive lifestyle factors were expected to be independently associated with lowered psychological distress and greater life satisfaction. We also expected an additive effect; in that the more healthy lifestyle choices an individual reported, the lower the psychological distress and the greater the life satisfaction reported by that participant would be.

## Method

### Procedure

This study was conducted as part of the Bochum optimism and mental health studies (BOOM-studies), which aim to investigate risk and protective factors of mental health in representative and student samples with cross-sectional and longitudinal assessments across different cultures. Presented data were collected between November 2012 and February 2013 through three professional opinion research institutes. Four different assessment methods were used: face-to-face interviews, telephone interviews, online survey, and a mixed-method-approach that allowed individuals to participate either online or via set-top box. All analyses and results presented in this study are controlled for their data assessment methods. Participants were either recruited via telephone or were registered members of an online panel. Trained professional interviewers conducted the telephone and personal interviews with computer assistance. The online and mixed-method data were assessed through self-administered surveys. Depending on the data assessment method, participants gave their informed consent written or orally after being informed about anonymity and voluntariness of the survey. Participants received no financial compensation. Representativeness for the German adult residential population, based on the register-assisted census data from 2011 regarding age, gender and education, was ensured via systematized sampling procedures. This procedure included the next-birthday-method, resulting in an equal chance for all household members of being selected for the telephone or face-to-face interview. The Ethics Committee of the Faculty of Psychology of the Ruhr-Universität Bochum approved the study.

### Participants

In total, 7,937 participants completed the survey. Participants were between 18 and 99 years old (*M* = 50.18, *SD* = 16.58) and 54.2% (*n* = 4,160) were females. About half of the participants (51.2%, *n* = 4,064) identified themselves as married, 29.5% (*n* = 4,064) were single and 19.0% (*n* = 1,504) separated, divorced or widowed. The four subsamples with different data assessment methods varied in size between 23.6% (*n* = 1,870) for face-to-face interviews and 25.7% (*n* = 2,039) for online participants. Table 1 provides an overview of the sample characteristics, including gender, marital status, educational level and data assessment method.

**Table 1:** **Sample characteristics regarding sociodemographic variables**

**Age (** ***M*** **,** ***SD*** **)**	***M*** **= 50.18,** ***SD*** **= 16.58**	
	***n*** ^**a**^	**%**
Gender		
Women	4,160	54.2
Men	3,777	48.8
Marital status		
Married	4,064	51.2
Single	2,331	29.5
Separated, divorced, widowed	1,504	19.0
Education		
Secondary school qualification	2,103	26.8
Secondary school certificate	2,825	36.1
High school diploma	1,380	17.6
University degree	1,524	19.5
Assessment method		
Face-to-face	1,870	23.6
Telephone	2,007	25.3
Online	2,039	25.7
Mixed method	2,021	25.5
Total	7,937	100.0

*Note*. ^a^Numbers vary due to missing data.

### Measures

#### Lifestyle behaviours

##### Frequency of physical activity

Frequency of physical activity was assessed using one item rated on a scale ranging from 0 (none) to 3 (more than 4 times a week): “Do you exercise regularly? If yes, with what intensity have you exercised in the last 12 months?” Single-item measures of physical activity are characterized by an acceptable reliability and construct validity compared to objective measurement methods (Milton et al. 2011).

##### Frequency of mental/cultural activity

Frequencies of mental/cultural activities were assessed using one item rated on a scale ranging from 0 (none) to 3 (more than 4 times a week): “Do you regularly engage yourself in a mental activity, and if yes with what intensity have you done it in the last 12 months?”.

##### Alcohol consumption

Frequency of alcohol consumption was assessed using one item: “How often do you drink alcohol?” Answer categories were never, once a month, 2 to 4 times a month, 2 to 3 times a week and 4 times a week and more. While there is an on-going controversy about the validity of self-reported alcohol consumption compared to objective data, recent studies conclude that self-report can be a reliable estimate for alcohol consumption, especially in low to moderate drinkers (Gmel and Rehm 2004). To account for possible non-linear associations between alcohol drinking frequency and mental health (as described in Rodgers et al. 2000) a quadratic polynomial was added to the statistical analyses.

##### Smoking

Current smoking was assessed using one item: “Do you smoke regularly?” Answer categories were ‘no’, ‘yes, sometimes’ and ‘yes, regularly’. For the present analyses the two latter categories were combined into ‘yes’, which was coded as 1. ‘No’ was coded as 2.

##### Body mass index

Body mass index (BMI) was calculated from weight and height as weight divided by height squared (kg/m^2^). Height and weight were assessed via self-report. Self-reported measurements of height and weight have been found to be very reliable, with the exception of highly obese individuals. For this group a slight underestimation of weight has been reported (Gorber et al. 2007). Previous research indicated impaired mental health in underweight and obese individuals (e.g., Molarius et al. 2009), and thus a quadratic polynomial of body mass index was also added to the analyses.

##### Circadian and social rhythms

Circadian and social rhythms were assessed using the Life Rhythms Scale (Margraf, Lavallee, Zhang & Schneider, unpublished manuscript), which includes 10 items measuring an individual’s perceived life rhythm regarding sleep, meals, wake-up time and social contacts on a scale ranging from 1 (very regularly) to 6 (very irregularly). The first scale, “circadian rhythm”, consists of six items and includes statements about regularity of meals, going to bed and getting up on weekdays and weekends. The second scale, “social rhythm”, consists of four items and describes the regularity of social contacts with colleagues or friends on weekdays and weekends. In the present study, Cronbach’s Alpha was good with α = .84 for circadian rhythm and acceptable with α = .73 for social rhythm. Validity evidence comes from data indicating that the full scale is related to physical health, consistent with past research on rhythmicity and certain aspects of mental health (Margraf et al., unpublished manuscript).

#### Protective lifestyle score

To evaluate the combined effects of healthy lifestyle behaviours, we constructed a protective lifestyle behaviour (PLB) score, as such scores have been used to examine the combined influence of different lifestyle factors on mental or physical health (Harrington et al. 2010; Khaw et al. 2008). In this study, the PLB score was calculated via aggregation of all assessed lifestyle behaviours. On this account, lifestyle behaviours were dichotomized into two variables; one reflecting a rather healthy lifestyle, the other reflecting a relatively unhealthy lifestyle behaviour. Therefore, if a participant did not meet the minimum threshold he/she was assigned a 0 for the particular lifestyle behaviour, and if the participant met the minimum, he/she was assigned a 1. The threshold was defined with regard to the existing literature on dose–response relationships between lifestyle and mental health (Hamer et al. 2009) and with regard to our own analyses. Healthy lifestyle choices with respect to the PLB score included engaging in physical exercise or mental/cultural activity at least once a month, being non-smoker, moderation in alcohol consumption (once a month to three times per week), reporting a BMI between 18.5 and 29.9 (normal- or overweight, but not underweight or obese) and having a relatively regular social and circadian rhythm compared to others (median-split). Scores were then summed across variables. Summary scores ranged from zero (least healthy lifestyle) to seven (most healthy lifestyle) indicating the number of protective lifestyle behaviours shown by the participant.

#### Mental health questionnaires

##### Satisfaction with life

Life satisfaction was assessed with the Satisfaction With Life Scale (SWLS; Diener et al. 1985), a self-report questionnaire designed to assess the judgmental component of personal wellbeing with five items rated on a scale ranging from 1 (strongly disagree) to 7 (strongly agree). Summing across items yields a total score ranging from 5 to 35, with a cut-off of 19 for at least average life satisfaction. Research indicates that SWLS has high convergent and discriminant validity (Pavot and Diener 2008). In the present sample Cronbach’s Alpha was α = .89.

##### Depression, anxiety, and stress

The negative emotional states of depression, anxiety, and stress over the last seven days were assessed with the Depression Anxiety Stress Scales – 21 (DASS-21; Henry and Crawford 2005), a short version of the Depression Anxiety Stress Scales (Lovibond and Lovibond 1995). The DASS-21 consists of 21 items, 7 for each subscale, rated on a 4-point Likert scale ranging from 0 (did not apply to me at all) to 3 (applied to me very much, or most of the time). Summing across subscales yields three total scores from 0 to 21, with clinical cut-offs of 10 for depression (DASS-D), 6 for anxiety (DASS-A), and 10 for stress (DASS-S). Individuals that met this threshold were considered “symptomatic”. DASS-21 is a widely used instrument with good psychometric properties (Shea *et al*. 2009). Cronbach’s Alphas in the present sample were α = .90, α = .83 and α = .89 for the DASS-D, DASS-A and DASS-S, respectively.

#### Sociodemographic variables

Gender, age, educational level, marital status and data assessment method were assessed and controlled for in the following analyses.

### Data analyses

Data were analysed using SPSS (Version 21). Multiple hierarchical linear regression analyses were conducted to examine the relationship between lifestyle behaviours and mental health outcomes. We entered the sociodemographic variables in the first step, and added the seven separate lifestyle behaviours, supplemented by quadratic predictors for alcohol frequency and body mass index, in the second step. This procedure allowed for a determination of the variance explained in mental health by the lifestyle factors above and beyond the sociodemographic variables. To assess the combined effect of lifestyle on mental health, hierarchical logistic regressions were used to calculate odds ratios (OR) with 95% confidence intervals for the likelihood of being satisfied with life or feeling none to low psychological symptoms. The outcome variables were dichotomized into non-symptomatic and symptomatic. Again, control variables were included in the first step, followed by the protective lifestyle behaviour (PLB) score as the predictor in the second step. The reference category used was 0 to 2 protective lifestyle behaviours. Missing data were handled using a multiple imputation procedure.

## Results

### Sample characteristics

Table 2 displays the sample characteristics regarding lifestyle variables and mental health outcomes for women and men. Women described a higher frequency in mental/cultural activities compared to men, while men reported more frequent alcohol consumption. Women exhibited a lower body mass index, a more regular circadian rhythm as well as a less regular social rhythm compared to men. Average life satisfaction scores for both genders were slightly below 25, falling between the categories “average” and “high” life satisfaction (Diener et al. 1985). Significant gender differences in psychological distress were also found, with higher ratings for depression, anxiety, and stress in women. Gender differences were minimal to small (*d* < .20) for all factors, except alcohol consumption, which approached a medium effect size (*d* = .47) with men drinking significantly more often. Further details are displayed in Table 2.

**Table 2:** **Descriptive variables and t-tests of lifestyle behaviours and mental health measures for women and men**

		**Women**			**Men**			***t*** **(** ***df*** **)**	***CI*** **(** ***95*** **%)**	***d***
**Lifestyle behaviours**	**Range**	***n*** ^**a**^	***M***	***SD***	***n*** ^**a**^	***M***	***SD***			
Physical activity	0-3	4,118	1.85	1.39	3,739	1.85	1.39	−0.08 (7855)	−0.06 0.06	.00
Mental/cultural activity	0-3	4,124	2.61	1.36	3,741	2.44	1.39	5.70 (7863)***	0.12 0.24	.12
Alcohol frequency	0-3	4,137	1.52	1.20	3,755	2.11	1.30	−21.02 (7890)***	−0.65 -0.54	.47
Smoking	1-2	4,158	1.73	0.44	3,773	1.73	0.45	0.65 (7929)	−0.01 0.03	.00
Body mass index	14-67	2,811	25.90	5.64	2,874	26.88	4.72	−7.11 (5683)***	−1.25 -0.71	.19
Circadian irregularity	6-36	4,150	15.14	6.72	3,763	15.64	6.90	−3.30 (7911)**	−0.81 -0.21	.07
Social irregularity	4-24	2,937	14.31	5.08	2,576	13.83	4.86	3.57 (5511)***	0.22 0.74	.10
Mental health										
Life satisfaction	5-35	4,131	24.60	6.34	3,750	24.76	6.31	−1.12 (7879)	−0.44 0.12	.03
Depression	0-21	4,121	3.41	4.12	3,736	3.15	4.00	2.88 (7855)**	0.08 0.44	.06
Anxiety	0-21	4,103	2.52	3.30	3,717	2.35	3.22	2.38 (7818)*	0.03 0.32	.05
Stress	0-21	4,121	5.54	4.47	3,738	4.98	4.18	5.65 (7857)***	0.36 0.75	.13

*Note*. *p < .05; **p < .01; ***p < .001.

^a^Numbers vary due to missing data.

### Individual lifestyle behaviours and mental health

Table 3 displays bivariate correlations among lifestyle behaviours, life satisfaction, depression, anxiety, and stress. The lifestyle behaviours correlated significantly and in the expected direction with each other (i.e. healthy behaviours in one domain were correlated with healthy behaviours in other domains), with effects in the minimal to small range. The mental health measures were also inter-correlated; life satisfaction being negatively associated with depression, anxiety, and stress, which furthermore all correlated positively with each other with medium to large effect sizes.

**Table 3:** **Pearson correlation coefficients among lifestyle behaviours and mental health**

		**1**	**2**	**3**	**4**	**5**	**6**	**7**	**8**	**9**	**10**	**11**	**12**	**13**
1	Physical activity	1	.23^***^	.11^***^	-.05^***^	.13^***^	-.13^***^	-.09^***^	-.04^***^	-.21^***^	.21^***^	-.13^***^	-.10^***^	-.07^***^
2	Mental/cultural activity		1	.08^***^	.02^*^	.06^***^	.01	-.02	-.05^***^	-.09^***^	.14^***^	-.13^***^	-.08^***^	-.08^***^
3	Alcohol frequency (linear)			1	.11^***^	-.04^**^	-.04^**^	-.07^***^	.03^**^	-.07^***^	.08^***^	-.09^***^	-.10^***^	-.03^**^
4	Alcohol frequency (quadratic)				1	.00	.03^*^	-.01	-.03^**^	.08^***^	-.04^***^	.04^**^	.05^***^	.02
5	Smoking (yes/no)					1	.02^*^	-.02	-.14^***^	-.00	.11^***^	-.10^***^	-.06^***^	-.07^***^
6	Body mass index (linear)						1	.53^***^	.04^**^	.08^***^	-.07^***^	.06^***^	.09^***^	.05^***^
7	Body mass index (quadratic)							1	.04^**^	.05^***^	-.11^***^	.10^***^	.11^***^	.07^***^
8	Circadian irregularity								1	.19^***^	-.15^***^	.18^***^	.13^***^	.14^***^
9	Social irregularity									1	-.21^***^	.19^***^	.13^***^	.15^***^
10	Life satisfaction										1	-.56^***^	-.39^***^	-.42^***^
11	Depression											1	.75^***^	.70^***^
12	Anxiety												1	.64^***^
13	Stress													1

Note. *p < .05; **p < .01; ***p < .001.

Lifestyle behaviours correlated significantly with all mental health measures; these effect sizes were also mostly small. Associations were in the expected direction with supposedly healthier behaviours relating to higher life satisfaction as well as to lower depression, anxiety, and stress. The quadratic term of alcohol frequency was negatively correlated with life satisfaction, and positively correlated with levels of depression and anxiety, indicating greater mental health for moderate drinkers compared to abstainers and very frequent drinkers. A similar pattern was found for the squared BMI term, which was also negatively correlated with life satisfaction and positively correlated with all three measures of psychological distress.

The results of the hierarchical regression analyses are presented in Table 4. Step 1 included the control variables gender, age, educational level, marital status, and assessment method. Step 2 added the lifestyle behaviours as predictors. Greater frequency of mental/cultural activities, non-smoking and regular social and circadian life rhythms were associated with greater mental health regarding all four outcome measures. Physical activity was related to greater life satisfaction as well as lower depression and anxiety scores. The significant squared terms of alcohol drinking frequency indicated lower mental health levels in all four outcome measures for abstainers and very frequent drinkers. The significant quadratic term of BMI indicated lower levels of life satisfaction and greater levels of depression and anxiety in underweight and obese individuals. The greater the BMI, the greater the levels of reported symptoms of stress, even though only significant on a 5% level. All lifestyle predictors together explained a significant proportion of variance in life satisfaction (*R* ^*2*^ = .06), depression (*R* ^*2*^ = .07), anxiety (*R* ^*2*^ = .04), and stress (*R* ^*2*^ = .04). Effect sizes for all predictor x outcome combinations were small.

**Table 4:** **Hierarchical multiple linear regression analyses predicting mental health from lifestyle behaviour variables**

	**Life satisfaction**		**Depression**		**Anxiety**		**Stress**	
	**ß**	***CI*** **(** ***95*** **%)**	**ß**	***CI*** **(** ***95*** **%)**	**ß**	***CI*** **(** ***95*** **%)**	**ß**	***CI*** **(** ***95*** **%)**
Step 1: Sociodemographic variables								
Gender	0.01	−0.02 0.03	−0.03*	−0.05 -0.01	−0.03*	−0.05 -0.01	−0.07***	−0.09 -0.05
Age	0.02	−0.01 0.04	−0.07***	−0.10 -0.05	−0.01	−0.04 0.02	−0.14***	−0.17 -0.12
Education	0.12***	0.10 0.15	−0.13***	−0.15 -0.10	−0.13***	−0.15 -0.10	−0.07***	−0.09 -0.04
Marital status^a^ Reference: married								
Divorced	−0.15***	−0.17 -0.12	0.09***	0.06 0.11	0.04***	0.02 0.07	−0.01	−0.03 0.02
Single	−0.15***	−0.17 -0.12	0.09***	0.06 0.12	0.05***	0.02 0.07	−0.01	−0.03 0.02
Method^a^ Reference: online								
Mixed	0.07***	0.04 0.09	−0.09***	−0.11 -0.06	−0.10***	−0.13 -0.07	−0.05**	−0.08 -0.02
Face-to-face	0.09***	0.06 0.11	−0.19***	−0.22 -0.16	−0.21***	−0.24 -0.19	−0.17***	−0.20 -0.14
Telephone	0.27***	0.25 0.30	−0.21***	−0.24 -0.18	−0.18***	−0.20 -0.15	−0.12***	−0.15 -0.10
*R* ^*2*^	.10		.06		.04		.05	
Step 2: Sociodemographic variables and lifestyle behaviours								
Gender	0.01	−0.02 0.03	−0.03*	−0.05 0.00	−0.02	−0.05 0.00	−0.08***	−0.10 -0.06
Age	0.02	−0.01 0.04	−0.06***	−0.08 -0.03	0.00	−0.03 0.03	−0.15***	−0.17 -0.12
Education	0.07***	0.04 0.09	−0.07***	−0.10 -0.05	−0.08***	−0.11 -0.06	−0.03**	−0.06 -0.01
Marital status^a^ Reference: married								
Divorced	−0.13***	−0.15 -0.11	0.07***	0.04 0.09	0.02	0.00 0.05	−0.02	−0.04 0.00
Single	−0.14***	−0.17 -0.12	0.08***	0.05 0.10	0.03*	0.01 0.06	−0.02	−0.04 0.01
Method^a^ Reference: online								
Mixed	0.10***	0.07 0.12	−0.13***	−0.70 -3.00	−0.14***	−0.17 -0.11	−0.09***	−0.11 -0.06
Face-to-face	0.09***	0.07 0.12	−0.20***	−0.11 -7.00	−0.22***	−0.25 -0.19	−0.18***	−0.21 -0.15
Telephone	0.23***	0.20 0.25	−0.17***	−0.70 -3.00	−0.15***	−0.18 -0.12	−0.10***	−0.13 -0.07
Physical activity	0.10***	0.07 0.12	−0.05***	−0.07 -0.02	−0.04*	−0.06 -0.01	−0.02	−0.04 0.01
Mental/cultural activity	0.06***	0.03 0.08	−0.09***	−0.11 -0.07	−0.07***	−0.09 -0.05	−0.07***	−0.10 -0.05
Alcohol frequency (linear)	0.04***	0.02 0.06	−0.06***	−0.08 -0.03	−0.08***	−0.10 -0.05	0.01	−0.02 0.03
Alcohol frequency (quadratic)	−0.05***	−0.07 -0.03	0.05***	0.03 0.07	0.06***	0.04 0.08	0.04***	0.02 0.06
Smoking (yes/no)	0.06***	0.03 0.08	−0.05***	−0.07 -0.03	−0.03*	−0.05 0.00	−0.03**	−0.06 -0.01
Body mass index (linear)	−0.04**	−0.07 -0.02	0.01	−0.01 0.04	0.03*	0.00 0.06	0.04*	0.00 0.07
Body mass index (quadratic)	−0.01*	−0.02 0.00	0.01**	0.01 0.02	0.02*	0.00 0.03	0.01	0.00 0.01
Circadian irregularity	−0.08***	−0.10 -0.06	0.12***	0.09 0.14	0.10***	0.08 0.13	0.09***	0.07 0.11
Social irregularity	−0.15***	−0.17 -0.13	0.14***	0.12 0.17	0.08***	0.06 0.10	0.14***	0.11 0.16
*R* ^*2*^	.16		.13		.08		.09	

Note. *p < .05; **p < .01; ***p < .001.

^a^Categorial predictors were dummy-coded; each category presented is tested against all other categories.

### Protective lifestyle behaviour (PLB) score and mental health

Participants varied in the number of their protective lifestyle behaviours. Only 0.1% exhibited no protective behaviours, while 0.8% and 3.8% showed either one or two of such. Therefore, these participants were taken together for the following analyses. A PLB score of three was obtained by 11.3%, a score of four by 20.2%, a score of five by 28.6%, a score of six by 24.3%, and a score of seven by 10.9% of the participants. Table 5 displays the results of the hierarchical logistic regression analyses of the protective lifestyle behaviour scores on dichotomized mental health measures. Overall, the higher the PLB score, the higher the odds of being in the categories of low psychological distress and high life satisfaction. For example, odds ratios of being non-symptomatic in depression were 2.21 (95% CI = 1.63 – 2.98) for individuals with 4 PLBs and 12.07 (95% CI = 7.09 – 20.56) for persons with 7 PLBs compared to the reference category (0 to 2 PLBs). In other words, the odds of reporting substantial symptoms of depression were about 12 times higher in individuals with 0 to 2 PLBs than in individuals with 7 PLBs. A similar pattern was found for the other outcome variables and is shown in Table 5.

**Table 5:** **Hierarchical multiple logistic regression analyses predicting mental health from protective lifestyle behaviour score**

	**High life satisfaction** ^**a**^ **SWLS > 19**		**Low depression** ^**a**^ **DASS depression scale < 10**		**Low anxiety** ^**a**^ **DASS anxiety scale < 6**		**Low stress** ^**a**^ **DASS stress scale < 10**	
	***Odds ratio***	***CI 95*** **%**	***Odds ratio***	***CI*** **(** ***95*** **%)**	***Odds ratio***	***CI*** **(** ***95*** **%)**	***Odds ratio***	***CI*** **(** ***95*** **%)**
Step 1: Sociodemographic variables								
Gender	1.01	0.90 1.13	1.14	0.97 1.33	1.13	0.99 1.29	1.28***	1.13 1.45
Age	1.00*	1.00 1.01	1.02***	1.01 1.02	1.00	1.00 1.01	1.02***	1.02 1.03
Education	1.27***	1.20 1.34	1.38***	1.27 1.49	1.35***	1.26 1.43	1.17***	1.10 1.24
Marital status^b^ Ref.: single								
Married	2.11***	1.81 2.46	1.64***	1.31 2.05	1.28**	1.07 1.52	1.15	0.96 1.38
Divorced	1.24*	1.02 1.49	1.09	0.83 1.43	0.99	0.80 1.24	1.18	0.94 1.47
Method^b^ Ref.: online								
Face-to-face	1.50***	1.28 1.75	3.52***	2.78 4.46	3.14***	2.60 3.80	2.76***	2.28 3.32
Mixed	1.45***	1.24 1.70	1.84***	1.49 2.27	2.03***	1.70 2.44	2.09***	1.74 2.50
Telephone	3.47***	2.89 4.16	3.43***	2.71 4.35	2.61***	2.18 3.14	1.61***	1.37 1.90
Step 2: Sociodemographic variables and protective lifestyle behaviour score								
Gender	1.01	0.90 1.14	1.14	0.97 1.34	1.14	1.00 1.29	1.28***	1.13 1.46
Age	1.01***	1.00 1.01	1.02***	1.01 1.02	1.00	1.00 1.01	1.02***	1.02 1.03
Education	1.16***	1.10 1.23	1.24***	1.14 1.34	1.26***	1.18 1.34	1.07*	1.01 1.14
Marital status^b^ Reference: single								
Married	1.95***	1.66 2.28	1.46**	1.17 1.84	1.17	0.98 1.4	1.05	0.87 1.26
Divorced	1.17	0.97 1.42	1.02	0.78 1.33	0.95	0.76 1.18	1.12	0.90 1.40
Method^b^ Reference: online								
Face-to-face	1.52***	1.29 1.79	3.64***	2.86 4.62	3.22***	2.66 3.90	2.81***	2.33 3.40
Mixed	1.59***	1.35 1.87	2.07***	1.67 2.56	2.21***	1.84 2.65	2.30***	1.92 2.77
Telephone	2.88***	2.39 3.47	2.74***	2.15 3.49	2.23***	1.86 2.69	1.35**	1.14 1.59
Protective Lifestyle Behaviour Score (PLB)^b^ Ref: 0-2								
3 PLB	1.26	0.96 1.65	1.73**	1.24 2.39	1.56**	1.12 2.18	1.73***	1.28 2.35
4 PLB	2.06***	1.59 2.65	2.21***	1.63 2.98	1.85***	1.41 2.43	2.06***	1.56 2.71
5 PLB	3.09***	2.39 3.99	3.37***	2.51 4.53	2.73***	2.09 3.56	3.09***	2.37 4.04
6 PLB	4.25***	3.27 5.52	5.32***	3.86 7.35	3.26***	2.43 4.36	4.03***	3.04 5.34
7 PLB	6.57***	4.70 9.18	12.07***	7.09 20.56	5.52***	3.71 8.22	6.36***	4.40 9.20

Note. *p < .05; **p < .01; ***p < .001.

^a^Outcome variables were dichotomized in symptomatic and non-symptomatic categories according to literature.

^b^Categorial predictors were dummy-coded; each category presented is tested against the reference category.

## Discussion

The primary objective of the present study was to evaluate the relationship between multiple everyday lifestyle behaviours and mental health in a large representative community sample in Germany. Our analyses indicated that mental health is indeed associated with different lifestyle behaviours. Physical activity, mental/cultural activity, frequency of alcohol consumption, smoking, body mass index, circadian and social rhythms were all significantly associated with life satisfaction, depression, anxiety, and stress. The importance of physical exercise, moderate alcohol consumption, non-smoking and healthy body weight for mental health supported the findings from past research and extended them to the German adult population. Additionally, the independent contribution of mental/cultural activities and regular life rhythm to mental health is particularly interesting, as findings until now have been somewhat inconclusive regarding social rhythm and a spectrum of disorders other than bipolar depression. The present data complements the current state of research in this area (Cuypers et al. 2012).

Combining multiple lifestyle behaviours into one protective lifestyle behaviour score indicated an additive relationship between healthy behaviours and mental health. There was a strong trend of decreasing psychological distress corresponding with an increasing number of protective lifestyle behaviours. The more health promoting behaviours reported, the better the reported mental health outcomes were. These results were in line with previous research that utilized protective lifestyle scores and examined their relation to depression (Harrington et al. 2010). The present study adds substantial information about the application of PLB scores not only as an important correlate of affective disorders, but also of anxiety and stress, as well as of life satisfaction, a positive facet of psychological wellbeing. Also in line with other large-scale surveys on different lifestyle factors and mental health outcomes, the proposed associations were mostly statistically significant, although the overall effect sizes were small (Herring et al. 2010; Molarius et al. 2009).

Another interesting finding concerns the meaningful associations between all four mental health outcomes and some sociodemographic factors. While age and gender were significant predictors of depression and stress, educational level showed significant associations with all outcomes and marital status was associated with life satisfaction, depression, and anxiety. Our analyses showed that higher education and being married were associated with better mental health as compared with lower education or being single or divorced. Effect sizes for these sociodemographic variables were comparable to effect sizes of the proposed lifestyle predictors. Meta-analytic results suggest that this is not an incidental finding, but reflects actual associations. An aggregation of 51 studies revealed that individuals with low socioeconomic status, often also operationalized through the level of education, had higher odds of being depressed (odds ratio = 1.18, *p* < .001) compared to persons with high socioeconomic status (Lorant et al. 2003). The association between marital status and mental health has also been documented, with lower rates of anxiety and depression prevalent in married individuals compared or divorced/widowed persons (Afifi et al. 2006; Holt-Lunstad et al. 2008). Generalizability and external validity were assured via the utilization of a large population-based representative sample and validated measures for both lifestyle and mental health. The observed effect of data assessment methods on mental health outcomes might seem surprising, but it also reflects existing research. The substantial differences between different data assessment methods and mental health were not expected, but seem to be very relevant not only as a limitation to our findings, but also as an important research question. The presented data definitely suggests careful consideration of data assessment methods, as differences between online, telephone and face-to-face interviews were obvious, with online surveys resulting in higher reporting of psychological distress compared to personal or telephone interviews [unpublished data]. To this point, it is unclear if the higher levels of distress reported in self-administered surveys reflect an over reporting of symptoms by the participants, or indicate a greater openness to revealing substantial psychological distress due to the higher perceived anonymity of the setting.

### Limitations

Several methodological limitations that challenge internal validity should be considered. First and foremost, the lack of longitudinal data is a shortcoming that prevents us from examining the proposed bidirectional relationship between lifestyle and mental health. Additionally, the reliance of self-report and the lack of objective data might have led to more socially desirable responding. While circadian and life rhythms, life satisfaction, depression, anxiety, and stress were assessed with carefully constructed psychometric instruments, alcohol consumption, physical, cultural/mental activity, smoking and body mass index were each assessed with only one item each. Although these or similar items have been used in several previous studies (Milton et al. 2011), and have demonstrated sufficient reliability and validity, memory biases and socially desirable responding may have extra effects on these items.

### Implications

Future projects should include longitudinal data to examine the possible bidirectional relationship between lifestyle and mental health. Cross-cultural comparisons are necessary to determine whether the findings are similar across different cultures. Further, lifestyle factors should be assessed with more precision in order to evaluate the dose–response relationship and to minimize recall biases. The use of diary-based instruments is promising for this purpose, especially for physical or mental/cultural activities and alcohol consumption. Diary methods could also be relevant supplements in the assessment of life rhythms. Despite the high reliability of the Life Rhythms Scale, it remains unclear how subjectively participants interpret the regularity of their own lives. Implementation of a more precise instrument, like the Social Rhythm Metric (Monk et al. 1991), may increase the validity of the analysis of the relationship between social regularity and mental health. Additional use of objective data, such as activity assessment via pedometer, might also increase internal validity. Further differentiation between mental activities, like reading books and more social, cultural activities, like going to the movies or playing music in a band, is necessary to control for potential overlap between these constructs and to distinguish between rather creative or receptive activities (Cuypers et al. 2011).

## Conclusion

Our findings support and supplement the existing literature on lifestyle and mental health, indicating that lifestyle behaviours, including physical and mental activity, alcohol consumption, smoking, life rhythm and body mass index are individually predictive of life satisfaction, depression, anxiety, and stress. The number of healthy lifestyle choices also predicts lower psychological distress and greater life satisfaction. Healthy lifestyle behaviours seem to have an additive effect on mental health.
