# Erratum to: Lifestyle choices and mental health: a representative population survey

**DOI:** 10.1186/s40359-016-0106-7

**Published:** 2016-01-22

**Authors:** Julia Velten, Kristen L Lavallee, Saskia Scholten, Andrea Hans Meyer, Xiao-Chi Zhang, Silvia Schneider, Jürgen Margraf

**Affiliations:** Faculty of Psychology, Department of Clinical Psychology and Psychotherapy, Mental Health Research and Treatment Center, Ruhr-Universität Bochum, Massenbergstrasse 9-13, D-44787 Bochum, Germany; Department of Psychology, University of Basel, Basel, Switzerland

Due to a technical error, a 21-item version of the Depression Anxiety and Stress Scales (DASS-42; Lovibond & Lovibond, 1995) which is not identical to the DASS-21 validated by Henry and Crawford (2005) was used in our study [1]. Ten items of the DASS version that was used in our study [1] were identical to the items of the original DASS-21, the remaining eleven items were part of the DASS-42, but not of the DASS-21 by Crawford and Henry (2005) [2]. In order to put the results of our paper into perspective, we have conducted an additional study that aimed to investigate the comparability of our 21-item version with the original DASS-21 that has been validated by Henry and Crawford (2005) [2].

A total of 1,031 individuals (47.9 % male) participated in a web-based survey, which included the DASS-42. Mean age of the participants was 48 years (*SD* = 15.26).

Comparisons between the original DASS-21 and the version that was used in our study revealed that both scales were highly similar. Correlations between the original DASS-21 subscales and the subscales of the version that was used in our study were very high (*r* = .97, *p* < .001 for depression, *r* = .93, *p* < .001 for anxiety, and *r* = .94, *p* < .001 for stress). Differences in the mean scores for all subscales were negligible to small (Cohen’ s d was 0.06 for depression, 0.07 for anxiety, and 0.24 for stress). The internal consistency of our version (α = .93 for depression, α = .85 for anxiety, and α = .90 for stress) was also comparable to the original DASS-21 (α = .93 for depression, α = .86 for anxiety, and α = .91 for stress).

Upon referring to our article [1], please acknowledge that we have used a 21-item version of the DASS-42 that is similar but not identical with the scale that has been validated by Crawford and Henry (2005) [2].
